# Antibacterial Activities of *Agaricus bisporus* Extracts and Their Synergistic Effects with the Antistaphylococcal Drug AFN-1252

**DOI:** 10.3390/foods13111715

**Published:** 2024-05-30

**Authors:** Milica Jankov, Vincent Léguillier, Uroš Gašić, Jamila Anba-Mondoloni, Maja Krstić Ristivojević, Aleksandra Radoičić, Ivica Dimkić, Petar Ristivojević, Jasmina Vidic

**Affiliations:** 1 Innovative Centre of the Faculty of Chemistry Ltd., Studentski trg 12-16, 11158 Belgrade, Serbia; 2 Micalis, AgroParisTech, National Research Institute for Agriculture, Food and the Environment (INRAE), University Paris-Saclay, UMR 1319, 78350 Jouy en Josas, France; 3 Department of Plant Physiology, Institute for Biological Research “Siniša Stanković”—National Institute of the Republic of Serbia, University of Belgrade, Bulevar despota Stefana 142, 11108 Belgrade, Serbia; uros.gasic@ibiss.bg.ac.rs; 4 Department of Biochemistry, Centre of Excellence for Molecular Food Sciences, University of Belgrade, Studentski trg 12-16, 11000 Belgrade, Serbia; krstic_maja@chem.bg.ac.rs; 5 Department of Biochemistry and Molecular Biology, Faculty of Biology, University of Belgrade, Studentski trg 16, 11158 Belgrade, Serbia; ivicad@bio.bg.ac.rs; 6 Department of Analytical Chemistry, Centre of Excellence for Molecular Food Sciences, University of Belgrade, Studentski trg 12-16, 11000 Belgrade, Serbia; ristivojevic@chem.bg.ac.rs

**Keywords:** button mushroom, acetonic extracts, ethanolic extracts, MRSA, bi-therapy

## Abstract

*Agaricus bisporus*, commonly known as the button mushroom, has attracted attention for its biological properties, including antimicrobial activities. Here, we evaluated the efficacy of ethanolic and acetonic extracts from white and brown *A. bisporus* against different bacterial strains, including antibiotic-resistant strains. Bioautography and principal component analysis identified the most active antibacterial compounds for each of the tested bacteria and indicated the main markers responsible for the strain-specific effects. In addition, the mushroom extracts demonstrated a synergistic impact when combined with the antistaphylococcal antibiotic AFN-1252.

## 1. Introduction

While antibiotics have undoubtedly played a crucial role in improving human health and life expectancy, there has been a major increase in bacterial infections that fail to respond to the antibiotics currently in clinical use [1]. The most important driver of antimicrobial resistance is the inadequate and unjustified utilization of antibiotics, both in humans and animals, which in turn leads to the selection, survival, and spread of resistant bacterial strains [2]. Among these, multidrug-resistant (MDR) pathogens, which cannot be treated with standard therapies, are of special concern. Although the World Health Organization recognizes that antibiotics are becoming increasingly ineffective, only a few novel drugs are currently in the pipeline. There is thus an urgent need for new antimicrobial strategies [1,2,3]. The process of developing novel antibiotics is highly challenging because of a wide variety of technical, financial, and regulatory hurdles. Furthermore, as soon as any new antibiotic is introduced, bacteria evolve new mechanisms of antimicrobial resistance. This was the case, for example, with recently developed antimicrobials targeting the fatty acid synthesis (FASII) pathway in bacteria. In addition to developing mutations in the FASII target genes, bacteria began to incorporate exogenous fatty acids in their membrane lipids, thus dispensing with the need for FASII [4].

To preserve the efficiency of existing antibiotics and limit the spread of resistant bacterial strains, drugs must be employed judiciously and their use combined with a variety of preventive strategies [3,5]. In this context, natural products are being explored as promising alternatives for the treatment and prevention of bacterial infections. Mushrooms are known for their numerous pharmacological effects, including antioxidant, anti-inflammatory, antidiabetic, antihyperlipidemic, hepatoprotective, anticancer, and prebiotic properties, as well as their protective effects against bacterial infections [6,7]. Previous studies on mushroom extracts have suggested strong antibacterial activity of extracts from *Lactarius deliciosus*, *Sarcodon imbricatus*, *Tricholoma portentosum*, *L. sulphureus*, *Pleurotus ostreatus*, and certain *Basidiomycota* [8,9,10]. Moreover, pleuromutilin derivatives from basidiomycetes have been approved by the Food and Drug Administration (FDA) for human use [11]. In general, though, much remains to be discovered about the potential therapeutic applications of mushrooms.

Edible mushrooms are a substantial source of healthy nutrients, and their consumption has increased consistently in recent years [7]. Apart from their nutritional value, edible mushrooms may have functional properties and thus represent an outstanding reservoir of compounds with bioactive and therapeutic properties [12]. One of the most widely consumed varieties is the button mushroom, *Agaricus bisporus*, which is appreciated for its low-calorie content, lack of saturated fat and cholesterol, and source of essential amino acids, vitamins, and fiber. *A. bisporus* has two color states when immature, including white and brown, and is not generally considered to have medicinal value. However, it has been found to contain polysaccharides, glycoproteins, triterpenoids, phytochemicals, phenolic compounds, and flavonoids [12], all of which strongly suggest that it may have some therapeutic potential. This hypothesis has been supported by several reports that *A. bisporus* exhibits antitumor, antioxidant, anti-inflammatory, and antimicrobial activities [13,14,15,16].

In order to further explore the potential of this easily culturable mushroom, here, we evaluated the antimicrobial activity of ethanolic and acetonic extracts from white and brown *A. bisporus* on non-pathogenic, pathogenic, and MDR bacterial strains. In addition, we evaluated the potential synergistic effects of combining *A. bisporus* extracts with the antistaphylococcal anti-FASII drug AFN-1252 in the treatment of multi-drug resistant *Staphylococcus aureus*.

## 2. Materials and Methods

### 2.1. Chemicals and Materials

Ethanol (96%) was purchased from Sani-hem (Novi Bečej, Serbia). Sodium dihydrogen phosphate, sodium hydroxide, 3-(4,5-dimethylthiazol-2-yl)-2,5-diphenyltetrazolium bromide (MTT), and Triton X-100 were purchased from Sigma-Aldrich (Steinheim, Germany). Nutrient agar slants were bought from Lab M (Bury, UK), and Brain Heart Infusion (BHI) broth was acquired from Oxoid Ltd. (Basingstoke, UK). Toluene was purchased from Zorka Pharma (Šabac, Serbia), ethyl acetate from Betahem (Belgrade, Serbia), and formic acid anhydride from Lach-Ner (Neratovice, Czech Republic). Luria Bertani (LB) broth, HPLC-grade methanol, and HPTLC silica gel 60 F 254 20 × 10 cm glass plates were purchased from Merck (Darmstadt, Germany). AFM-1252 and adult bovine serum were purchased from CliniSense (Nanterre, France). Fatty acids (C14:0, myristic acid; C16:0, palmitic acid; and C18:1, oleic acid) were solubilized in dimethyl sulfoxide (DMSO) as 100 mM stocks and used at the final equimolar concentration of 0.17 mM each (referred to later as eFAs). eFAs were purchased from Larodan Fine Chemicals (Stockholm, Sweden).

### 2.2. Extraction

*Agaricus bisporus* mushrooms were provided by the organic mushroom farm Ekofungi (Belgrade, Serbia). The mushrooms were washed, sliced, dried at 40 °C for 72 h, and ground in an electric grinder. Then, 5 g of each sample was ultrasonicated for 1 h with 50 mL of ethanol or acetone (1:10, *w*/*v*). Supernatants were obtained by centrifugation (10,000 rpm, 15 min) on a Thermo Scientific SL 16 centrifuge (Waltham, MA, USA) and then evaporated under reduced pressure. The obtained residues were dissolved in methanol to a final concentration of 25 mg/mL. The extracts were filtered through a 0.45 μm filter before use.

### 2.3. Total Phenolic Content and Protein Content

Total phenolic content (TPC) was determined using the Folin–Ciocalteu method [17]. Briefly, 0.5 mL of the extracts and 0.5 mL of ultrapure water were mixed with 2.0 mL of 10% Folin–Ciocalteu reagent. After 5 min, 2.5 mL of 7.5% sodium carbonate was added. The mixture was left to stand for 2 h, and the absorbance was measured at 765 nm. Gallic acid (20–100 mg/L) was used as a standard, and the results were expressed as mg gallic acid equivalent per mL of extract (mg GAE/mL). Total proteins were determined using a Bradford assay (Invitrogen, Nanterre, France). TPC and protein concentrations are presented as mean ± SD.

### 2.4. Agar Well Diffusion Method

Bacterial cells were cultured in LB broth at 17.7–21.7 McF density; suspensions were then diluted with PBS to ~5 McF. A 1 mL aliquot of diluted suspension was mixed with 50 mL nutrient agar and poured into a Petri dish (15 cm diameter). Mushroom extracts (25 mg/mL, 60 µL each) were transferred into the wells (10 mm diameter). The inoculated Petri dishes were placed in a refrigerator for 1 h to allow the compounds to diffuse into the agar, and then the dishes were incubated at 37 °C for 24 h. All experiments were performed in triplicate.

### 2.5. HPTLC Analysis

Mushroom extracts (25 mg/mL, 10 μL each) were applied to an HPTLC glass plate as 6 mm long bands using a Linomat 5 applicator (CAMAG, Muttenz, Switzerland). The separation of extract compounds was achieved using MF ethyl acetate–dichloromethane–formic acid–methanol in a ratio of 10:10:1:3 (*v*/*v*/*v*/*v*). A CAMAG Twin Trough Chamber was saturated with mobile phase vapor for 20 min, and the HPTLC plate was developed to a solvent front of 80 mm. The chromatogram was derivatized for visualization with a p-anisaldehyde solution using an immersion TLC chromatogram device (CAMAG) for 3 s at an immersion speed of 4.5 cm/s. After drying the plate in an oven at 100 °C, the compounds became visible as colorful bands on a white background. Images were taken under visible light using a Samsung S21 mobile phone (Samsung Electronics, Suwon-si, Republic of Korea) equipped with a camera of 64 MPs.

### 2.6. HPTLC Bioautography Assay

HPTLC bioautography assays were carried out with *Bacillus subtilis* (ATCC 6633), *Staphylococcus aureus* (ATCC 6538), methicillin-resistant *S. aureus* (ATCC 33591), and *Escherichia coli* (ATCC 35218). Bacterial strains were cultivated on nutrient agar slants at 37 °C for 24 h. Each well-grown culture was suspended in 5 mL of sterile physiological solution. From each cell suspension, 0.1 mL was used to inoculate 10 mL of Luria–Bertani (LB) broth and incubated overnight on a BioSan Orbital Shaker-Incubator ES-20 at 37 °C and 220 rpm. Bacterial suspensions were used for derivatization when the suspension turbidity reached 4.48 McF for *B. subtilis*, 4.91 McF for MRSA, 5.12 McF for *S. aureus*, and 4.62 McF for *E. coli.* The developed HPTLC chromatograms were immersed in bacterial suspensions for a few seconds and then incubated in a humidity chamber at 37 °C under aerobic conditions for 90 min, allowing the bacteria to grow on the plate surface. Antibacterial zones were visualized using a thermostatted 0.1% MTT solution in 0.1 M phosphate buffer, pH 7.2. In the case of the *E. coli* assay, 0.1 mL of Triton X-100 was added to the MTT solution. An additional 60-min incubation was performed, and positive reactions were noted, indicated by a color change in active white bands against a purple background. Images of the HPTLC chromatograms were captured under visible light using a Samsung S21 mobile phone (Samsung Electronics) equipped with a camera of 64 MPs.

### 2.7. LC/MS Metabolite Identification

LC-HRMS/MS (Thermo Scientific™ Vanquish™ Core HPLC system coupled to the Orbitrap Exploris 120 mass spectrometer, San Jose, CA, USA) was used to determine the metabolic profile of the mushroom extracts. The liquid chromatography system was equipped with a Hypersil GOLD™ C18 analytical column (50 × 2.1 mm, 1.9 μm particle size) thermostated at 40 °C. The injection volume was 5 μL, and the flow rate was constant at 300 μL/min. The compounds of interest were eluted with ultrapure water supplemented with 0.1% formic acid (A) and acetonitrile (MS grade) with 0.1% formic acid (B) as follows: 5% B in the first min; 5–95% B from 1 to 10 min; 95% B from 10 to 12 min; and 5% B until 15 min.

The Orbitrap Exploris 120 mass spectrometer was equipped with a heated electrospray ionization (HESI-II) source operating the negative ionization mode. Full scan MS were monitored from 100 to 1500 *m*/*z* with Orbitrap resolution set to 60,000 FWHM, while data-dependent MS2 experiments were conducted at an Orbitrap resolution of 15,000 FWHM. The normalized collision energy was set to 35% with an isolation width of 1.5 *m*/*z*. The dynamic exclusion time was set to 10 s with exclusion from a specific scan after 2 occurrences, and the intensity threshold was set to 1 × 105.

LC/MS data were evaluated using R Studio (version 2023.09.1, build 494) software. Peak picking was performed using the enviPick R package, and peak correspondence across samples was performed using the density method available in the xcms R package [18]. The identification of the metabolites was performed based on their chromatographic behavior and HRMS/MS2 data by comparison with standard compounds, when available, and literature data providing a tentative identification [19,20,21,22,23,24,25]. Data acquisition was carried out with the Xcalibur^®^ data system (Thermo Finnigan, San Jose, CA, USA).

### 2.8. Bi-Therapy Assay

The synergistic bactericidal activity of *A. bisporus* extracts and the antibiotic AFM-1252 was assessed against the two *S. aureus* strains (RN-4220 and the multidrug-resistant strain USA300-JE2). After overnight cultures in BHI broth, exponentially growing cells were obtained as subcultures in SFA medium (BHI broth supplemented with myristic acid, palmitic acid, oleic acid, and adult bovine serum at 10% final concentration), with shaking at 100 rpm for 3 h. The bacterial solutions were then divided equally into 96-well plates to which AFN-1225 and/or mushroom extracts were added. Bacterial growth was monitored by measuring optical density at 600 nm (OD600) using a Tecan Spark^®^ (Tecan, Männedorf, Switzerland) at 37 °C every 15 min for 18 h. Bacterial growth was also observed in a control sample in only the SFA medium. All experiments were performed at least in triplicate.

### 2.9. Image Processing and Multivariate Analysis

Images of HPTLC chromatograms were processed with ImageJ (https://imagej.net/Downloads, version1.47q (accessed on 5 September 2023)). Original images were converted to 8-bit black and white format (Image/Type/8-bit) and the background was subtracted (Process/SubtractBackground/1000 pixels). Each extract lane was marked with the rectangular selection tool, and the gray value dependence of the distance (pixels) along the line (RF) was generated with the Analyze/Plot Profile option. All obtained RF data values were then used for principal component analysis (PCA) following preprocessing techniques such as variable alignment [correlation optimized warping (COW)], normalization, and mean centering to achieve an equal impact of all separated compounds on the PCA model. PCA was carried out using the PLS ToolBox (v.6.2.1, www.eigenvector.com/software/pls_toolbox.htm (accessed on 5 September 2023.)) in MATLAB software (v. 7.12.0, R2011a).

## 3. Results and Discussion

### 3.1. Extracts of A. bisporus and the Well Diffusion Assay

The extraction yields, total phenolic content, and total protein content of ethanolic (E) and acetonic (A) extracts of white (W) and brown (B) *A. bisporus* mushrooms are given in Table 1. Phenolic compounds are a large group of fungal metabolites with a wide range of biological effects, including antibacterial and antioxidant activity [26]. In addition to these, a variety of mushroom proteins have been shown to have antifungal, antiviral, and antibacterial properties [26,27,28]. Of the two extractants, a higher extraction yield was obtained using ethanol compared with acetone. Surprisingly, acetonic extracts of white mushrooms had the lowest extraction yield but the highest total protein content (Table 1). This suggests the increased content of compounds with high polarity (such as proteins and peptides) or insoluble compounds with higher molecular weight in white *A. bisporus.*

**Figure 1:**
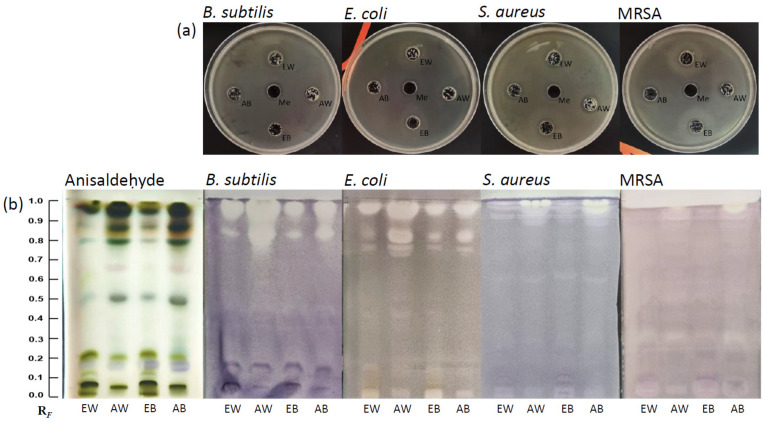
Agar well diffusion assay showing the antibacterial effects of *A. bisporus* extracts (**a**). HPTLC fingerprints of mushroom extracts after derivatization with anisaldehyde reagent ((**b**), left panel) and HPTLC bioautograms of mushroom extracts incubated with four bacterial strains ((**b**), right panel). EW, ethanolic extract of white *A. bisporus*; EB, ethanolic extract of brown *A. bisporus*; AW, acetonic extract of white *A. bisporus*; AB, acetonic extract of brown *A. bisporus*.

A well diffusion assay was used for the primary assessment of the antibacterial activity of extracts towards *S. aureus*, *E. coli*, *B. subtilis*, and methicillin-resistant *S. aureus* (MRSA), with methanol as the reference (Figure 1a). The four extracts exhibited antibacterial effects, but all strains displayed growth within the inhibitory zone. As expected, bacterial growth was entirely blocked by methanol. The results of this assay indicated that the complex mixtures of mushroom extracts possessed antibacterial properties, but bacterial colonies resistant to these extracts were still able to grow within the inhibitory zones.

### 3.2. HPTLC Fingerprinting and HPTLC Bioassays

HPTLC was used to screen the different *A. bisporus* extracts and compare their metabolite profiles. To visualize the HPTLC fingerprint, HPTLC chromatograms were derivatized using anisaldehyde, which is a non-selective reagent that enables the derivatization of various compounds including phenols, terpenes, steroids, and sugars [29]. All four extracts from *A. bisporus* showed similar HPTLC profiles (Figure 1b, left panel), suggesting they are similar in their molecular composition. However, acetonic extracts of both white and brown mushrooms (AW and AB) demonstrated a pronounced band at RF 0.70, along with strongly expressed bands at RF 0.50, 0.64, 0.79, and 0.86. From the ethanolic extracts (EW and EB), highly concentrated bands were visible at RF 0.07 and 0.21. High-intensity bands at RF 0.94 and 0.97 were observed in all extracts.

To further investigate the antimicrobial properties of the extracts, we employed HPTLC–bioautography to assess the individual compounds separated on the plate. HPTLC bioassays on *B. subtilis, S. aureus*, MRSA, and *E. coli* highlighted many active constituents, represented as white bands against a purple background (Figure 1b). The bands exhibiting antimicrobial activity were more pronounced in acetonic extracts, with the highest intensity bands observed with the AW, while the lowest activity was found in the EB extract for all four bacterial strains. In all four biochromatograms, the bands exhibiting the strongest antibacterial activity were located at RF 0.86 and 0.94. Interestingly, the biochromatograms obtained for pathogenic *S. aureus* and MRSA had active bands of lower intensities compared with those of the two non-pathogenic strains, *E. coli* and *B. subtilis*. In addition, the biochromatograms for *B. subtilis* and *E. coli* showed weak-intensity bands at RF 0.05 and 0.11 that were not observed with the *S. aureus* strains.

Taken together, the HPTLC fingerprint profiles of *A. bisporus* extracts suggest that there may be several antibacterial compounds that inhibit the growth of both Gram-positive and Gram-negative bacteria, but their efficacy seems to be strain-specific.

### 3.3. Principal Component Analysis

**Figure 2:**
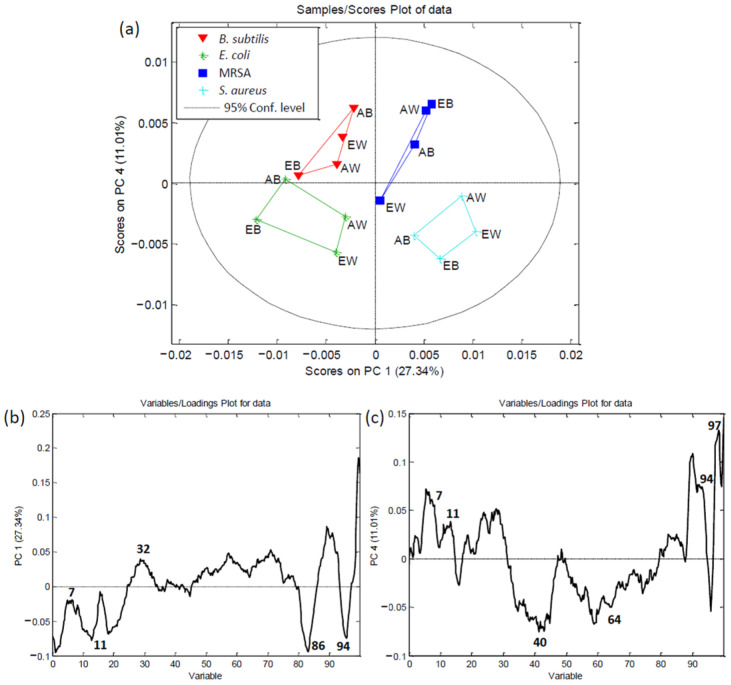
Principal component analysis (PCA) based on antibacterial HPTLC bioautograms: Sample/score plot of data (**a**), Variable/loading plot of data for PC1 (**b**), and Variable/loading plot of data for PC4 (**c**). EW, ethanolic extract of white *A. bisporus*; EB, ethanolic extract of brown *A. bisporus*; AW, acetonic extract of white *A. bisporus*; AB, acetonic extract of brown *A. bisporus*.

Principal component analysis (PCA) was performed on the data obtained from the HPTLC chromatograms to compare the antibacterial activities against the tested bacterial strains. The first six main components described 88.56% of the total variability in the data (PC1–27.34%, PC2–26.31%, PC3–11.91%, PC4–11.01%, PC5–7.49%, and PC6–4.49%). Figure 2a depicts the separation of the different extracts based on their antibacterial activity towards a given bacterial strain, as represented using the principal components PC1 and PC4.

In the PC plot, there was a clear pattern of grouping based on the bacterial strain used in each assay (Figure 2b,c); the chromatograms obtained using the four strains clustered into four different groups along the PC1 axis. Data from *E. coli* and *B. subtilis* formed a cluster on the left side, while pathogenic *S. aureus* and MRSA were clustered on the right side of the PC score plot. Then, components affecting the growth of Gram-positive bacteria were separated from those inhibiting *E. coli*. Within the Gram-positive biochromatograms, the objects were not distributed uniformly but displayed a clear separation from each other. PC1 was negatively correlated with bands at RF 0.11, 0.86, and 0.94. In addition, the band at RF 0.32 and 0.90 distinguished the MRSA assays from those using other strains along the PC1 axis. Finally, strongly expressed bands at RF 0.11, 0.86, and 0.94 negatively affected separation along the PC1 axis. Along the PC4 axis, the clustering of objects from the biochromatograms for *B. subtilis* and MRSA on the upper side was influenced by the active bands at RF 0.7, 0.11, 0.94, and 0.97. The objects obtained from the biochromatograms for *E. coli* and *S. aureus* were positioned on the lower part of the PC4 axis, and this separation was influenced by the bands at RF 0.40 and 0.64.

In this way, PCA was able to effectively distinguish the bioactive components influencing the growth of various bacterial strains, with clear separation observed along the PC1 and PC4 axes. The separated bands played a crucial role in the differentiation process, particularly influencing the distribution of Gram-positive and Gram-negative bacteria. This analytical approach highlights the potential of PCA for streamlining the identification of antibacterial agents, thereby aiding in the targeted treatment of bacterial infections.

### 3.4. LC/MS Profiling of Metabolites

**Figure 3:**
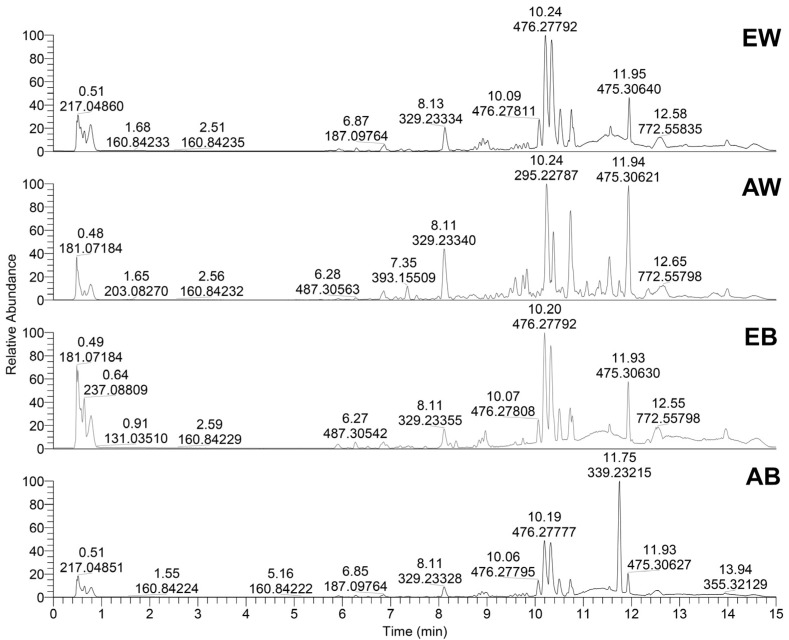
Base peak chromatograms of the ethanolic extract of white *A. bisporus*, EW; acetonic extract of white *A. bisporus*, AW; ethanolic extract of brown *A. bisporus*, EB, and acetonic extract of brown *A. bisporus*, AB. Identified compounds are provided in Supplementary Table S1.

UHPLC-Orbitrap MS characterization of extracts of white and brown *A. bisporus* resulted in the detection of 41 metabolites (Table 2). The identified compounds can be divided into six different groups as follows: (1) phenolic acids (7 compounds), (2) amino acids (10 compounds), (3) fatty acids (8 compounds), (4) steroids (3 compounds), (5) peptides (2 compounds), and (6) 11 compounds classified as other metabolites. Interestingly, the metabolic profiles differ significantly between white and brown mushrooms and also between their acetonic and ethanoic extracts, as shown in their base peak chromatograms (Figure 3). However, 9,10,13-Trihydroxy-11-octadecenoic acid, 8-Hydroxy-9,12-octadecadienoic acid, and unknown steroid 2 were identified as major compounds found in all extracts. In addition, 9,10,13-Trihydroxy-11-octadecenoic acid was found in higher amounts in white mushrooms compared with brown mushrooms. Unknown steroid 2 was detected as a major metabolite in AW, EW, and EB, while AB extract contain a low peak of the mentioned metabolite. Supplementary Table S1 shows the peak areas of compounds identified in all four *A. bisporus* extracts.

### 3.5. Synergistic Bi-Therapy

AFN-1252, an inhibitor of the FASII pathway, is used as a last-resort treatment against *S. aureus* infection. However, this species has been shown, in both animals and humans [45], to evolve bypassing mutations that enable the utilization of exogenous fatty acids to compensate for FASII inhibition [4]. Because *S. aureus* variants that are adapted to AFN-1252 may constitute a reservoir for new, potentially untreatable infections, it is necessary to find ways to preserve the efficacy of this treatment. One possible method is combining AFN-1252 with synergistic treatments that will potentiate its efficiency against staphylococcal infections and help to prevent chronic disease.

**Figure 4:**
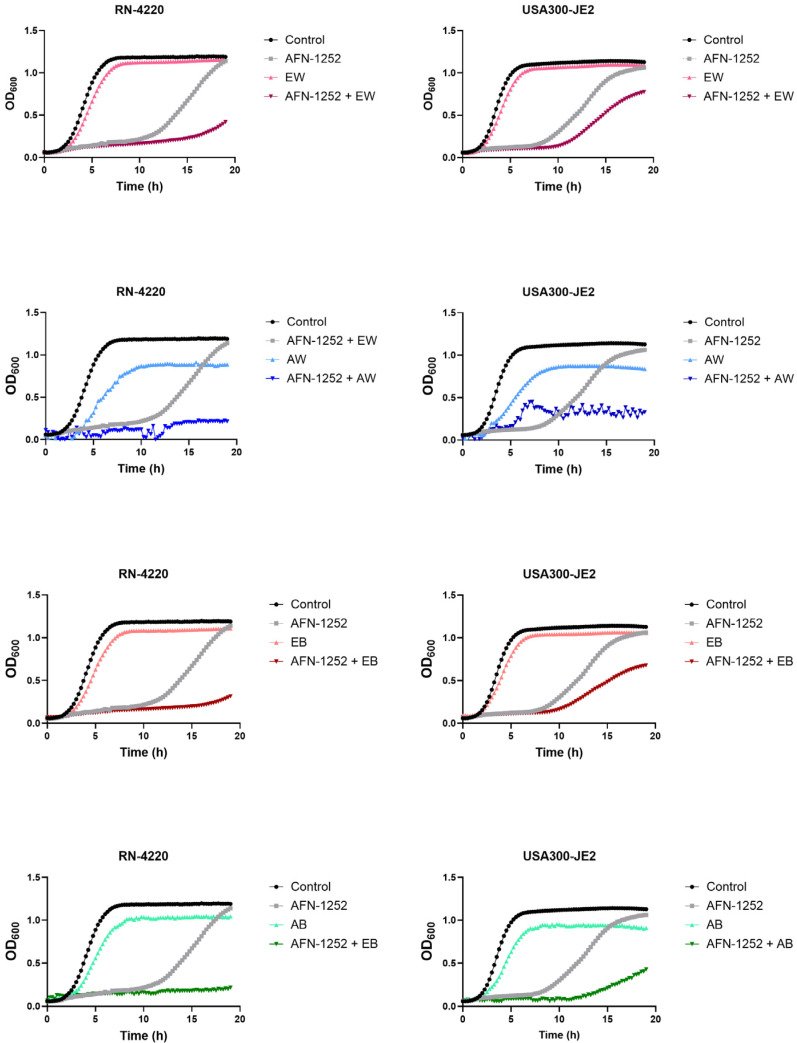
*A. bisporus* extracts exhibit synergistic effects when applied with the anti-FASII antibiotic AFN-1252. Bacterial cells were treated with 6 µL of the indicated extract (25 mg/mL each) and/or 0.5 µg/mL AFN-1252 for 20 h; the optical density at 600 nm was then measured. EW, ethanolic extract of white *A. bisporus*; EB, ethanolic extract of brown *A. bisporus*; AW, acetonic extract of white *A. bisporus*; AB, acetonic extract of brown *A. bisporus*.

To determine whether the mushroom extracts have synergistic effects with AFN-1252, we treated cultures of *S. aureus* in the exponential growth phase with individual extracts, AFN-1252, or mixtures of extracts and AFN-1252. Growth was monitored in BHI broth supplemented with fatty acids to enable bacteria to bypass the effects of AFN-1252, i.e., to incorporate fatty acids in membrane lipids and thus dispense with the need for FASII. As shown in Figure 4, in this medium, both *S. aureus* strains (RN-4220 and USA300-JE2 (MRSA)) were able to adapt to AFN-1252 and began exponential growth after a 10 h latency phase. The four mushroom extracts only weakly inhibited the proliferation of bacterial cells, with ethanolic extracts of white *A. bisporus* (EW) causing a transient 2 h latency in growth (Figure 4). However, when AFN-1252 and ethanolic extracts were applied together, the anti-FASII latency period was strikingly longer than when the antibiotic was applied alone, and in the cultures exposed to both AFN-1252 and the AW extract, bacterial growth was completely inhibited. Interestingly, the combination of AFN-1252 and the AB extract completely suppressed the growth of strain RN-4220 but only partially that of MRSA (Figure 4).

The observed synergistic effect between the mushroom extracts and AFN-1252 suggests that *A. bisporus* extracts may have the potential for use as adjuvants to antibiotic therapy, which could possibly lead to the development of highly efficient treatments against *S. aureus* infection. Given that *S. aureus* bypasses FASII inhibition by integrating fatty acids into its membrane, the observed synergistic effect may be the result of the mushroom extract somehow blocking the incorporation of fatty acids. In addition, mushrooms are known to contain many unsaturated fatty acids (such as C18:2n6, C20:1, C20:2, C20:4n6, C22:2, and C22:1n9) and long saturated chains (C23:0 and C24:0) [46] that are antibacterial agents [47,48]. It could also be that the observed effect is caused by the incorporation of these compounds into bacterial membranes to compensate for FASII inhibition. Our LC-HRMS/MS analysis confirmed the presence of multiple unsaturated fatty acids with 18C in *A. bisporus* (Table 2 and Supplementary Table S1). Future research will need to elucidate the exact means by which *A. bisporus* compounds exert their antibacterial effects in order to evaluate and optimize their potential for use in the prevention of the anti-FASII adaptation of methicillin-resistant pathogens like *S. aureus*.

## 4. Conclusions

Extracts from *A. bisporus* demonstrated notable inhibition of the growth of *B. subtilis*, *S. aureus*, *E. coli*, and methicillin-resistant *S. aureus*. Bioautography revealed that the extracts investigated here contained several antimicrobial compounds with strain-specific effects. In addition, PCA highlighted the main markers responsible for the discrimination between bacterial strains. Moreover, synergistic antimicrobial effects were observed when the mushroom extracts were combined with the antibiotic AFN-1252, especially the acetonic extract of white *A. bisporus*, which completely blocked the ability of *S. aureus* to bypass AFN-1252 activity. Based on these results, we hypothesize that the bioactive compounds in *A. bisporus* may disrupt bacterial cell walls or metabolic pathways, and thus enhance the potency of traditional antibiotics. Together, these findings highlight the potential of natural mushroom products as alternatives or supplements to current antimicrobial treatments amidst escalating antibiotic resistance.

## Figures and Tables

**Table 1:** Total phenolic compounds and total protein contents of button mushrooms obtained using two different extractants.

Extract	Yield of Extraction (mg/g)	Total Phenolic Content (mg/mL)	Total Protein Content (mg/mL)
EW	51.40 ± 5.5	0.51 ± 0.05	1.02 ± 0.3
EB	60.80 ± 5.2	0.49 ± 0.04	1.33 ± 0.12
AW	8.46 ± 0.65	0.54 ± 0.06	2.38 ± 0.15
AB	28.78 ± 5.33	0.29 ± 0.09	1.00 ± 0.23

EW, ethanolic extract of white *A. bisporus*; EB, ethanolic extract of brown *A. bisporus*; AW, acetonic extract of white *A. bisporus*; AB, acetonic extract of brown *A. bisporus*. Data are expressed as mean ± S.D. of duplicate measurements.

**Table 2:** LC/HRMS data for metabolites identified in *Agaricus bisporus* extracts.

No	Compound Name	*t*_R_, min	Molecular Formula, [M–H]–	Calculated Mass, *m/z*	Exact Mass, *m/z*	Δ ppm	MS^2^ Fragments, (% Base Peak)	Ref
***Phenolic acids***								
**1**	**Gallic acid**	0.68	C_7_H_5_O_5_^–^	169.01425	169.01429	−0.28	**125.02459** (100), 169.01451 (42)	[19]
**2**	**Dihydroxybenzoic acid**	1.12	C_7_H_5_O_4_^–^	153.01933	153.01941	−0.53	**109.02964** (100), 153.01941 (48)	[30]
**3**	**Hydroxybenzoic acid 1**	2.61	C_7_H_5_O_3_^–^	137.02442	137.02459	−1.25	**93.03468** (100), 137.02461 (29)	[31]
**4**	**Benzoic acid**	4.93	C_7_H_5_O_2_^–^	121.02950	121.02966	−1.31	**121.02971** (100)	[32]
**5**	**Caffeic acid**	5.25	C_9_H_7_O_4_^–^	179.03498	179.03513	−0.80	**135.04564** (100), 179.03488 (7)	[20]
**6**	***p*-Coumaric acid**	6.04	C_9_H_7_O_3_^–^	163.04007	163.04021	−0.89	**119.05042** (100)	[19]
**7**	**Hydroxybenzoic acid 2**	6.77	C_7_H_5_O_3_^–^	137.02442	137.02457	−1.08	**93.03466** (100), 137.02438 (68)	[31]
***Amino acids***								
**8**	**L-Threonine**	0.49	C_4_H_8_NO_3_^–^	118.05100	118.05109	−0.76	**74.02480** (100), 118.05112 (34)	[33]
**9**	**D-Asparagine**	0.50	C_4_H_7_N_2_O_3_^–^	131.04622	131.04629	−0.53	69.03466 (4), **87.04523** (100), 113.02460 (9)	[33]
**10**	**L-Glutamic acid**	0.52	C_5_H_8_NO_4_^–^	146.04588	146.04602	−0.94	**102.05613** (100), 128.03555 (55), 146.04623 (51)	[33]
**11**	**L-Aspartic acid**	0.53	C_4_H_6_NO_4_^–^	132.03023	132.03039	−1.18	71.01395 (41), **115.00387** (100	[33]
**12**	**D-Valine**	0.53	C_5_H_10_NO_2_^–^	116.07170	116.07186	−1.35	73.02954 (57), 99.00883 (6), **116.07183** (100)	[33]
**13**	**L-Pyroglutamic acid**	0.64	C_5_H_6_NO_3_^–^	128.03532	128.03542	−0.78	**128.03543** (100)	[33]
**14**	**L-Glutamyl-L-leucine**	0.65	C_11_H_19_N_2_O_5_^–^	259.12995	259.13007	−0.46	127.05146 (11), 128.03622 (50), **130.08778** (100), 197.13062 (22), 241.11896 (17)	[33]
**15**	**Tyrosine**	0.65	C_9_H_10_NO_3_^–^	180.06662	180.06673	−0.62	101.02451 (28), 119.05041 (51), 136.07693 (8), 163.04037 (92), **180.06688** (100)	[33]
**16**	**D-α-Aminoadipic acid**	0.65	C_6_H_10_NO_4_^–^	160.06153	160.06173	−1.25	**99.04529** (100), 101.02456 (40), 116.03542 (23), 143.03522 (10)	[21]
**17**	**D-Phenylalanine**	0.77	C_9_H_10_NO_2_^–^	164.07170	164.07182	−0.73	72.00916 (35), 91.05540 (6), 103.05539 (6), **147.04532** (100)	[21]
***Fatty acids***								
**18**	**9,10,13-Trihydroxy-11-octadecenoic acid**	8.13	C_18_H_33_O_5_^–^	329.23335	329.23352	−0.52	139.11314 (24), 171.10291 (67), 211.13429 (57), 229.14474 (42), **329.23380** (100)	[34]
**19**	**8-Hydroxy-13-oxo-9,11-octadecadienoic acid**	9.06	C_18_H_29_O_4_^–^	309.20713	309.20733	−0.63	113.09734 (70), 171.10291 (63), **195.10283** (100), 291.19696 (6)	[35]
**20**	**5,8-Dihydroxy-9,12-octadecadienoic acid**	9.58	C_18_H_31_O_4_^–^	311.22278	311.22298	−0.64	**171.10292** (100), 197.11871 (40), 211.13425 (86), 275.20178 (14), 293.21259 (80)	[34]
**21**	**Linolenic acid**	10.06	C_18_H_29_O_2_^–^	277.21730	277.21741	−0.37	134.03749 (34), 233.15533 (5), **277.21829** (100)	[36]
**22**	**8-Hydroxy-9,12-octadecadienoic acid**	10.25	C_18_H_31_O_3_^–^	295.22787	295.22793	−0.21	171.10284 (54), 195.1387 (9), **277.21747** (100), 295.22815 (70)	[37]
**23**	**16-Hydroxyhexadecanoic acid**	11.56	C_16_H_31_O_3_^–^	271.22787	271.22802	−0.56	225.22264 (83), **271.22809** (100)	[36]
**24**	**Linoleic acid**	11.93	C_18_H_31_O_2_^–^	279.23295	279.23309	−0.47	**279.23315** (100)	[36]
**25**	**2-Hydroxystearic acid**	12.32	C_18_H_35_O_3_^–^	299.25917	299.25930	−0.44	253.25391 (69), 281.24927 (2), **299.25934** (100)	[21]
***Steroids***								
**26**	**Unknown steroid 1**	9.80	C_29_H_47_O_7_^–^	507.33273	507.33273	−0.01	**387.29028** (100), 428.29233 (10)	NA
**27**	**Polyporusterone G**	10.73	C_28_H_43_O_5_^–^	459.31160	459.31166	−0.13	**325.25391** (100)	[22]
**28**	**Unknown steroid 2**	11.94	C_28_H_43_O_6_^–^	475.30651	475.30667	−0.33	97.02962 (11), 315.26965 (19), 369.31674 (13), **431.31708** (100)	NA
***Peptides***								
**29**	**Benzyl-2-[(1-hydroxy-4-methylpentan-2-yl)-carbamoyl]-pyrrolidine-1-carboxylate**	9.62	C_19_H_27_N_2_O_4_^–^	347.19763	347.19843	−2.31	166.01128 (13), 171.10306 (41), 211.13458 (19), 293.21323 (45), **311.22311** (100)	[23]
**30**	**2-Methyl-N-[N-[N-[(phenylmethoxy)-carbonyl]-isoleucyl]-leucyl]-alanine methyl ester**	10.20	C_25_H_38_N_3_O_6_^–^	476.27660	476.27778	−2.47	196.03815 (11), **279.23309** (100)	[23]
***Other metabolites***								
**31**	**Succinic acid**	0.50	C_4_H_5_O_4_^–^	117.01930	117.01948	−1.56	**73.02953** (100), 99.00886 (11), 117.01947 (45)	[38]
**32**	**Maleic acid**	0.52	C_4_H_3_O_4_^–^	115.00368	115.00381	−1.15	**71.01392** (100), 115.00381 (18)	[38]
**33**	**Oxaceprol**	0.73	C_7_H_10_NO_4_^–^	172.06153	172.06166	−0.77	96.04548 (26), 140.03525 (8), **172.06171** (100)	[38]
**34**	**Glutaric acid**	0.90	C_5_H_7_O_4_^–^	131.03498	131.03515	−1.30	**87.04521** (100), 113.02464 (7), 131.03514 (41)	[39]
**35**	**Adipic acid**	1.37	C_6_H_9_O_4_^–^	145.05063	145.05081	−1.20	83.05041 (6), **101.06094** (100), 145.05069 (29)	[40]
**36**	**Hexanoic acid**	6.44	C_6_H_11_O_2_^–^	115.07645	115.07658	−1.13	71.01392 (54), **115.07677** (100)	[41]
**37**	**Agaritine**	6.53	C_12_H_16_N_3_O_4_^–^	266.11463	266.11481	−0.68	74.05275 (19), 83.10571 (19), **128.03564** (100), 248.10542 (21)	[42]
**38**	**Indole-2-carboxylic acid**	6.56	C_9_H_6_NO_2_^–^	160.04040	160.04055	−0.93	116.05077 (67), **160.03983** (100)	[43]
**39**	**Azelaic acid**	7.14	C_9_H_15_O_4_^–^	187.09758	187.09772	−0.72	59.01388 (20), 87.00887 (31), 99.08166 (12), **125.09732** (100)	[44]
**40**	**Penipacid C**	7.34	C_10_H_9_N_2_O_4_^–^	221.05678	221.05707	−1.29	92.05076 (36), **136.04056** (100)	[30]
**41**	**Strobilactone A**	9.04	C_15_H_21_O_4_^–^	265.14453	265.14485	−1.21	203.14365 (6), **221.15497** (100), 247.13522 (23), 265.14548 (82)	[21]

*t*_R_—retention time (min); Δ ppm—mean mass accuracy; NA—not available.

## Data Availability

The original contributions presented in this study are included in this article. Further inquiries can be directed to the corresponding author.

## Supplementary Materials

The following supporting information can be downloaded at https://www.mdpi.com/article/10.3390/foods13111715/s1: Table S1: the peak areas of compounds identified in the *A. bisporus* extracts.
