# Supplementary material for: Antibacterial Activities of Agaricus bisporus Extracts and Their Synergistic Effects with the Antistaphylococcal Drug AFN-1252
Source: Foods. 2024 May 30;13(11):1715. doi: 10.3390/foods13111715 (PMC11172267; doi:10.3390/foods13111715)
Supplement: Supplementary file 1 [file foods-13-01715-s001.zip › foods-3019620-supplementary.pdf]

Supplementary data

Antibacterial activities of *Agaricus bisporus* extracts and their synergistic effects with the antistaphylococcal drug AFN-1252

Milica Jankov[1], Vincent Léguillier[2], Uros Gasic[3], Jamila Anba-Mondoloni[2], Maja Krstic Ristivojevic[4], Aleksandra Radoicic[1], Ivica Dimkic[5], Petar Ristivojevic[6], and Jasmina Vidic[2,*]

1 Innovative Centre of the Faculty of Chemistry Ltd., Studentski trg 12-16, 11158 Belgrade, Serbia.
2 University Paris-Saclay, INRAE, AgroParisTech, Micalis, UMR1319, 78350 Jouy en Josas, France.
3 Institute for Biological Research "Siniša Stanković", National Institute of Republic of Serbia, University of Belgrade, Bulevar despota Stefana 142, 11108 Belgrade, Serbia. U.G. uros.gasic@ibiss.bg.ac.rs
4 University of Belgrade, Department of Biochemistry, Centre of Excellence for Molecular Food Sciences, Studentski Trg 12-16, 11000 Belgrade, Serbia; M.K.R. krstic_maja@chem.bg.ac.rs
5 Faculty of Biology, University of Belgrade, Studentski trg 16, Belgrade, 11158, Serbia; I.D. ivicad@bio.bg.ac.rs
6 University of Belgrade, Department of Analytical Chemistry, Centre of Excellence for Molecular Food Sciences, Studentski Trg 12-16, 11000 Belgrade, Serbia; ristivojevic@gmail.com

*, Correspondence: J.V. jasmina.vidic@inrae.fr

Supplementary Table S1. The peak areas of compounds identified in the tested *Agaricus bisporus* extracts. Results are shown in triplicate for each extract (EW: P1_1-P1_3; AW: P2_1-P2_3; EB: P3_1-P3_3; AB: P4_1-P4_3)

No	Compound name	P1_1	P1_2	P1_3	P2_1	P2_2	P2_3	P3_1	P3_2	P3_3	P4_1	P4_2	P4_3
Phenolic acids													
**1**	**Gallic acid**				73214166	73345489	75311468				2989011	3033745	4088027
**2**	**Dihydroxybenzoic acid**	1041420	1522242	1724266	883366	1495177	2106988	2085839	3232582	3580704	3052888	4105353	1949895
**3**	**Hydroxybenzoic acid 1**	42868369	37473489	14761737	89122364	62687638	37715577	15418367	11401654	8313365	23168865	11431214	30491685
**4**	**Benzoic acid**				26180424	24588196	24565219				12736625	22526494	12666756
**5**	**Caffeic acid**				3139688	2865681	1203840	1675016	2721143	1043358	1500887	1514675	1528462
**6**	***p*-Coumaric acid**	4721478	4447488	4094398	7477653	7309799	7991095	3051343	2926667	2978827	2804861	3013169	3196634
**7**	**Hydroxybenzoic acid 2**	9155469	8692693	8924081				5112654	4853038	5584186			
Amino acids													
**8**	**L-Threonine**	59082053	60043006	33206519	59627184	58554330	64936668	42222874	42983537	44923813	41534197	29002047	38937153
**9**	**D-Asparagine**	12974368	15253012	11170218	8075362	9193121	8552793	10514235	10015406	10472667	11943261	6041526	12730785
**10**	**L-Glutamic acid**	74684902	73910484	73953513	7089437	7704593	7677163	55181592	59612103	58066773	58452116	58503681	58761453
**11**	**L-Aspartic acid**	6649361	2168142	5608396	2119938	2103809	1507441	551415	4236132	2131526	3290432	2572923	5822909
**12**	**D-Valine**	19246267	13140242	16548376	13320803	21710969	23364087	15354378	4555916	5088008	3292599	5517414	3934301
**13**	**L-Pyroglutamic acid**	820152554	721619680	791055981	86368839	107427836	104125516	545897954	560059221	553519180	784576092	778457183	783972507
**14**	**L-Glutamyl-L-leucine**	1766714	2663880	2344939	994050	1369269	1054075	2132720	2473472	2303096	1885927	1717222	2010517
**15**	**Tyrosine**	11601105	11279257	11052550	13440732	14682186	14172332	9615341	9428915	9042857	9894545	9456139	8848431
**16**	**D-α-Aminoadipic acid**	7904918	6981732	7492598	2756150	2370456	2578974	13235228	9995726	8188647	7223887	6704464	6679228
**17**	**D-Phenylalanine**	116784334	112716775	114829379	132758516	138701513	141282337	70985326	69435926	70930668	80790916	77657262	80965164
Fatty acids													
**18**	**9,10,13-Trihydroxy-11-octadecenoic acid**	580216680	567161770	582065605	1997773009	2006923503	2015573107	252968449	256057653	252592531	436246464	442477238	445085758
**19**	**8-Hydroxy-13-oxo-9,11-octadecadienoic acid**	59618510	59757502	88719950	299638580	299640961	301631261	35912703	55308742	32707889	47461387	57089496	61899121
**20**	**5,8-Dihydroxy-9,12-octadecadienoic acid**	266515024	269010727	267357003	1277600205	1249120281	1282430388	137652879	135718378	136879272	215994344	214864192	217569872
**21**	**Linolenic acid**	2961823	4847546	3296549	24585289	23877071	25022500	1733993	1411582	1267320	3847531	3671323	3782428
**22**	**8-Hydroxy-9,12-octadecadienoic acid**	837403019	828418680	821683316	4157501065	4195326910	4226896799	346357432	345413978	343544960	762075758	770089058	783190463

**23**	**16-Hydroxyhexadecanoic acid**	742953206	777083575	669585368	1625303380	1667162952	1684788166	378066612	303359478	342375123	482830210	503259821	490721312
**24**	**Linoleic acid**	37288492	38593371	38516903	417474781	432718770	433310388	16965580	16718809	16097177	32917312	34054685	34726693
**25**	**2-Hydroxystearic acid**	148125731	141817315	133523750	490463763	394389754	345881947	52660276	73872289	95056405	88708272	88837812	88499215
Steroids													
**26**	**Unknown steroid 1**	307984650	309314123	306880840	1558425759	1550950289	1560511785	154720524	156574839	157124812	242112567	246765985	249224033
**27**	**Polyporusterone G**	373785799	379272662	386980223	1229465468	1238945375	1296182945	453769855	444024528	433087568	287183359	289317289	294495439
**28**	**Unknown steroid 2**	1015760468	1008556723	838388613	3367050513	3425631479	3334591432	534671865	531651231	574895454	662530531	683460415	649172305
Peptides													
**29**	**Benzyl-2-[(1-hydroxy-4-methylpentan-2-yl)-carbamoyl]-pyrrolidine-1-carboxylate**	8353053	4884233	7381608	28157448	14393871	27415389	2324113	2100678	2303874	6911510	3226063	6160792
**30**	**2-Methyl-N-[N-[N-[(phenylmethoxy)-carbonyl]-isoleucyl]-leucyl]-alanine methyl ester**	2845085640	2838958030	2833277791	724789884	691352166	679145175	1366833827	1387027055	1394823333	2323186456	2358028945	2411362529
Other metabolites													
**31**	**Succinic acid**	206105410	195688636	199651964	152540676	152654807	152279007	122583809	126122362	120220153	133975003	137659353	142704298
**32**	**Maleic acid**	291476364	108257484	274454440	90901230	89863565	89127428	173982449	174674762	171299166	180070296	107086595	116260851
**33**	**Oxaceprol**	26786300	25501869	25786029	4169703	1216644	3744153	19807423	20197237	20630609	23713103	24262413	24002589
**34**	**Glutaric acid**	48466226	32587202	41180812	22134313	22623865	26829142	35848311	39082559	36770656	19507054	23334923	25379521
**35**	**Adipic acid**	12925676	20783218	12849948	12601742	14559998	12579382	12188660	10833733	9355584	7495763	7217614	7773911
**36**	**Hexanoic acid**	2749949	3173908	2822566	7789539	7857952	7671062	5088647	5434419	5559689	1837014	1849447	2126494
**37**	**Agaritine**	1077364	965322	1095710	485736	658821	991272				803252	799733	858396
**38**	**Indole-2-carboxylic acid**	224492	331225	296836	1481457	1590832	1421858	326551	373104	561781	269397	303711	235836
**39**	**Azelaic acid**	22666303	22594317	22039730	31581320	32389687	31359062	29897333	29155039	30985163	14658350	14713914	14079920
**40**	**Penipacid C**	1862232	1979459	1955328	901909	923446	963770	396368	391725	366417	728261	722137	672616
**41**	**Strobilactone A**				5877766	5771365	6460217				178678	200874	131709
